# Two-Needle Approach to Replenish an Empty Intrathecal Pump

**DOI:** 10.7759/cureus.95952

**Published:** 2025-11-02

**Authors:** Andrew Kent, Anterpreet Dua, Paramvir Singh, Ashraar Dua, Zhuo Sun

**Affiliations:** 1 Department of Anesthesiology and Perioperative Medicine, WellStar MCG Health, Medical College of Georgia, Augusta University, Augusta, USA

**Keywords:** intrathecal pump, needle aspiration, needle placement confirmation, pump pocket fill, pump refill, refractory pain, reservoir

## Abstract

Intrathecal pumps are devices developed for targeted central nervous system drug delivery in patients with severe, intractable pain who have failed traditional management, and in patients with secondary spasticity from an underlying condition refractory to oral medications. Typically, providers attempt to replenish the medication(s) of the reservoir before it is empty, with the residual volume being emptied prior to the refilling process. This is done in order to ensure verification of needle placement, since an empty reservoir can hamper needle placement verification. The inaccurate delivery of analgesic medication into the tissue surrounding an intrathecal pump, or "pocket fill," is a potentially life-threatening complication of pump management. This case report introduces a simple technique of using a second confirmatory needle along with the original refill needle in a patient whose pump reservoir has been empty for an extended period.

## Introduction

Intrathecal drug delivery systems are favorable modalities for the treatment of chronic non-cancer and cancer pain, as well as spasticity that has failed traditional management. This is evidenced by the shift in clinical practice whereby intrathecal drug delivery is used earlier in the treatment pathway, especially as it relates to chronic pain management [1]. This delivery technique, which consists of a battery-operated pump that is surgically implanted in subcutaneous tissue and dispenses medication(s) from its reservoir through a tunneled catheter directly into the intrathecal space, reduces systemic exposure and achieves the desired effect with much lower doses than required with oral or parenteral routes [2]. This, in turn, minimizes systemic side effects such as sedation [3]. To ensure continued treatment and benefit and avoid medication withdrawal symptoms, patients undergoing this treatment must receive periodic refills at frequencies specific to their condition and therapeutic regimen [2]. Withdrawal symptoms have been reported as approximately one event per 20.1 pump-years in baclofen-administering intrathecal pumps, with rates as high as 24% in a cohort of patients with implantable SynchroMed™ (Medtronic, Minneapolis, MN) pain pumps [4,5]. The refill procedure, which involves the insertion of a needle through the skin into the reservoir fill port, is typically done before the reservoir is empty to avoid pump failure. Moreover, once the needle has entered the reservoir, aspiration of the residual volume of medication is an additional method to ensure proper needle placement. Many clinicians compare this aspirated volume to a calculated residual volume by an external pump programmer [6,7]. Once the residual volume is aspirated, a new medication solution replaces the old within the reservoir [6]. Multiple mechanisms exist to confirm accurate needle placement within the reservoir, whether empty or not. These include template-guided localization, fluoroscopic guidance, ultrasound guidance, and, in complex cases, cone beam CT (CBCT) or high-resolution 3D CT imaging. Reliability is dependent on the method, with fluoroscopy and ultrasound guidance being the most accurate methods relative to template guidance for confirming needle placement, with advanced CT-based imaging reserved for complex cases [8,9]. Withdrawal of residual medication is a standard part of pump refill and is an auxiliary means of suggesting correct needle placement [6]. Thus, attempting to refill pumps with no residual medication increases the likelihood of erroneously injecting the solution into the surrounding tissue because of the inability to confirm accurate needle placement. This can lead to "pocket fill," whereby the medication is inserted in the subcutaneous tissue pocket surrounding the pump. These complications are caused by improper needle placement during the refill process and may result in immediate overdosing, withdrawal as a result of treatment for overdose, and delayed underdosing due to incomplete pump refill if not corrected [10]. Thus, determining the exact position of the pump and establishing secure access to the drug reservoir is critical to ensure patient safety. While various reports suggest that ultrasound or fluoroscopy is useful for port identification [9,11], refilling empty pumps places patients at risk of a pocket fill because the practitioner lacks the ability to aspirate fluid that would otherwise suggest proper needle placement. From May 1996 to September 2010, Medtronic Inc. received 351 reports worldwide of pocket fills, with eight being lethal. This makes pocket fills rare since thousands of pumps have been inserted [11]. With this concern, we developed a simple technique to confirm needle placement into an empty pump reservoir using two needles.

## Case presentation

**Figure 1:**
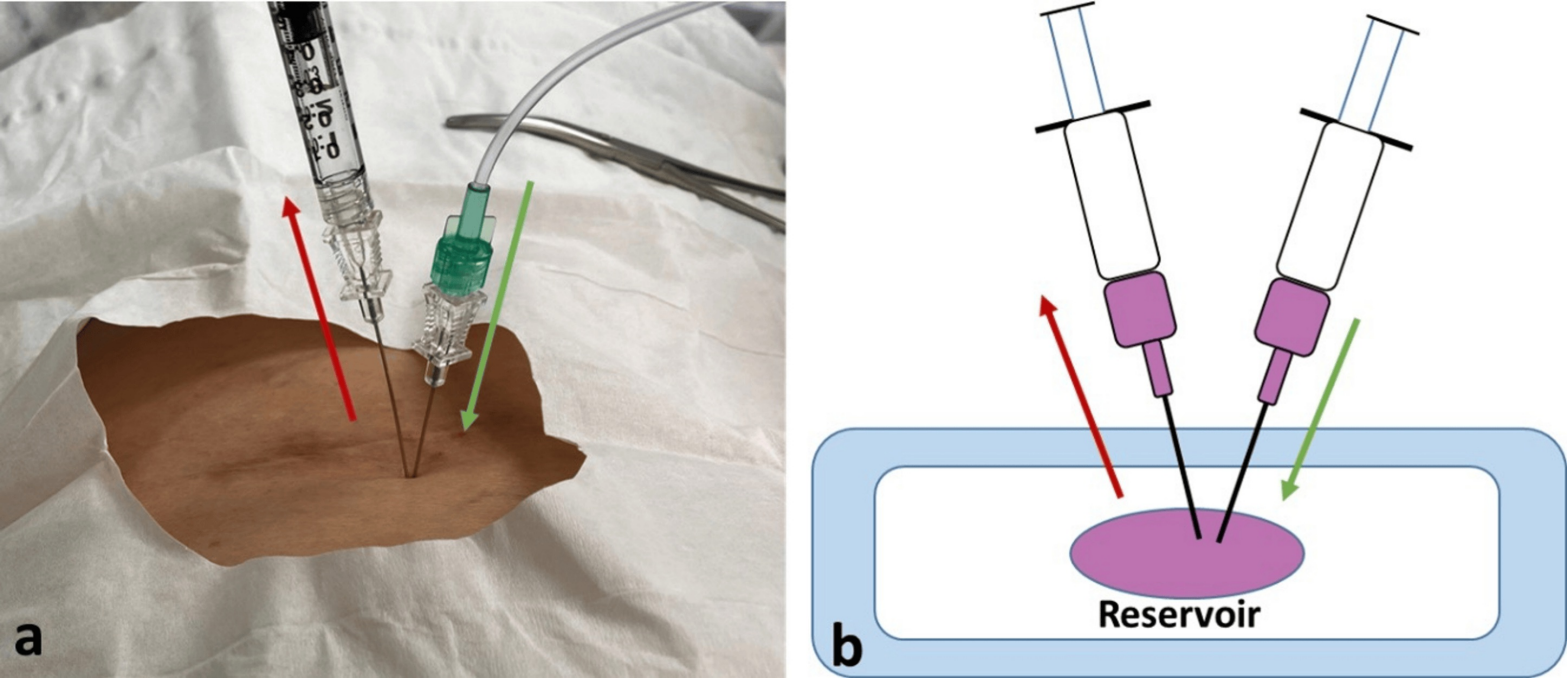
Photograph (a) and illustration (b) depicting the two-needle approach. Fluid is injected through one needle (green arrows) into an intrathecal pump reservoir. Flow of fluid through the other needle confirms placement (red arrows). Figure created using Microsoft PowerPoint (Microsoft Corp., Redmond, WA) drawing tool.

**Figure 2:**
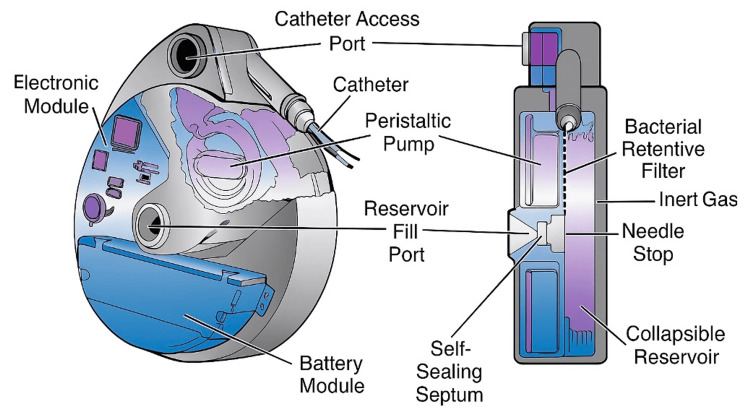
Diagram of the Medtronic SynchroMed™ II system and its components The Medtronic SynchroMed™ II system is composed of three chambers: an electronic module and battery, a peristaltic pump and expandable drug reservoir, and an inert gas. Image obtained from University Pain Centers; device manufactured by Medtronic [12].

The patient was a 79-year-old male with a surgical history of cervical and lumbar fusions and an implanted intrathecal pump for chronic lumbar radicular pain refractory to conservative management, who presented to the pain clinic to establish care. He had been unable to find a provider to refill his pump reservoir after recently moving between states, and as a result, his reservoir had been empty for at least two months. The patient did experience withdrawal symptoms; however, this was managed previously in the ER. The pump in this case is the Medtronic SynchroMed™ II System with the 20 mL reservoir, placed in the left anterior abdominal wall region. Hydromorphone was the medication utilized by the pump. Since the patient previously had adequate pain relief with his intrathecal pump, we decided to fill the pump and reinitiate intrathecal pump therapy (ITP). Next, we performed skin preparation and draping with an aseptic technique, including sterile gloves, mask, and chlorhexidine skin preparation within a sterile field. Next, we obtained pump reservoir access with the patient in the supine position under fluoroscopic guidance with a 20-gauge needle from the Medtronic 8551 Refill Kit. Since the reservoir had been empty for two months, there were no medications aspirated, and we couldn't utilize this method for accurate needle placement. To alleviate the concern for inaccurate needle placement, we developed a novel technique by connecting two syringes to two different needles to make a closed system by inserting the two needles in the pump reservoir refill port simultaneously. Although imaging can aid in the confirmation of correct needle placement, aspiration of fluid and comparison to calculated residual volume can also act as a second confirmatory measure. In this case, the tactile sensation of penetrating the rubber reservoir and the subsequent flow of fluid from the first needle into the second act as a confirmatory measure. With regard to over-accessing the pump, there is little to no concern since the manufacturer states the pump is designed with a self-sealing septum designed to withstand thousands of accesses. Understanding the design of the pump is crucial to understanding why backflow is observed through the second needle following the injection of medication through the first needle. The Medtronic's SynchroMed™ II system is composed of three chambers: an electronic module and battery, a peristaltic pump and expandable drug reservoir, and an inert gas. The reservoir is hermetically sealed and surrounded by an inert gas propellant. The constant pressure from the inert gas ensures that reservoir contents are always being pushed towards the peristaltic pump into the catheter. A preprogrammed rate is then input into the peristaltic pump to modulate how much drug is delivered through the catheter per unit of time. Consequently, if a second outlet was introduced into the reservoir, the pressure from the inert gas, along with the transiently increased pressure from drug insertion, would lead to drug efflux from the second outlet. This is observed because the pressure of the inert gas (in addition to the transient increase in pressure upon medication injection) is substantially higher than the resistance of the confirmatory syringe (which is calculated from the Hagen-Poiseuille equation). It should be noted that when applying the Hagen-Poiseuille equation, a modest injection rate of 0.1 mL/second through a 24-gauge needle with a length of 3.8 cm requires ≈126 mmHg of pressure to drive flow; 18-20G needles require even less. This is substantially lower than the reservoir pressure of the SynchroMed™ II implant, which is advertised as 155-261 mmHg by the manufacturer. Thus, immediate flow is observed upon medication insertion due to this pressure differential. Essentially, a closed hydraulic circuit is created. In our specific case, the refill needle was a 22-gauge, and the aspiration needle was a 24-gauge, both with a length of 3.8 cm. The prescribed pump medication, hydromorphone (4 mg/mL), was injected through the first needle, followed by subsequent visualization of this solution efflux from the second needle, which confirmed the accurate needle placement within the pump. As seen in Figure 1, this technique begins by inserting a refill and a confirmatory needle into the pump reservoir simultaneously. Then, the medication injection occurs through the refill needle (green arrows), and the visualization of smooth and effortless fluid outflow into the syringe through the confirmatory needle (red arrows) verifies the appropriate needle placement in the reservoir (Figures 1a-1b). Finally, the confirmatory needle was withdrawn prior to refilling the pump with the rest of the medication. Figure 2 highlights the Medtronic Pump and the compartments listed above that make the observed phenomenon possible. It should be noted that although the pump was primed, no bolus was planned; however, a low dose with continuous infusion at 40 mcg/day was employed. The next refill was hydromorphone (1 mg/mL) so that up-titration of the medication could occur with higher resolution.

## Discussion

The timely refilling of intrathecal pump reservoirs is essential, as delayed intervention could lead to life-threatening withdrawal symptoms. Because empty reservoirs do not have any residual medication to confirm accurate needle placement, refilling these devices with traditional techniques increases the risk of filling errors. The standard refill technique of intrathecal pump reservoirs utilizes either blind needle placement with a manufacturer template or imaging techniques such as ultrasound in conjunction with aspiration of residual medication to confirm needle placement and ensure accurate drug delivery. Since patients with empty pump reservoirs do not have residual medication for validating appropriate needle position, refilling these devices increases the risk of a pocket fill. Besides blind refilling practices, only one other method has been proposed to address empty intrathecal pump refilling: ultrasound-guided needle placement used in conjunction with post-refill volume verification with the pump’s fill level sensor [13]. Essentially, the pump’s fill level sensor can indicate if any medication injected did not settle in the pump, indicating pocket fill. Currently, there is no literature indicating the incidence rate of pocket fill using the above method. However, the literature does suggest that ultrasound-guided needle placement itself is more effective than blind or template-guided placement for complicated pump orientation [8]. Thus, while the above method is a reasonable approach, pocket fill cannot be determined until after the medication has already been injected and the volume sensor has had time to detect the medication levels. Moreover, some pumps do not have sensors. We propose a simple technique whereby two needles confirm needle placement in an empty pump, thus reducing the potential for pocket fill. Essentially, the second needle acts as a real-time pump volume sensor since concurrent medication efflux is noted with medication injection. Moreover, this effect would not be observed even if both needles were placed in the pump pocket, since the efflux is driven by the inert gas of the pump, which is not present in the pump pocket. This two-needle approach is a reliable alternative to refilling empty intrathecal pump reservoirs, as we have successfully performed six cases utilizing this technique with no observed complications. Further study using this confirmatory technique is still warranted, given the small number of cases and lack of long-term follow-up.

## Conclusions

Typical management of intrathecal pump reservoirs involves aspiration of fluid before refilling in order to ensure proper needle placement. However, this requires fluid to be present in the reservoir and thus is not a feasible option when the reservoir is empty. We propose a two-needle approach to safely confirm needle placement into an empty pump. This method is a quality alternative to replenishing the pump reservoir while avoiding potentially life-threatening overdose complications secondary to a pocket fill.
